# Comparison of norepinephrine with a combined non-catecholaminergic therapy in a porcine model of refractory cardiac arrest resuscitated with VA-ECMO

**DOI:** 10.1186/s40635-026-00895-4

**Published:** 2026-04-27

**Authors:** Thomas Klein, Vincent Elsensohn, Mathilde Martin-Schouler, Daniel Grandmougin, Thomas Raze, N.’Guyen Tran, Bruno Levy

**Affiliations:** 1 Service de Médecine Intensive et Réanimation Brabois, Université de Lorraine, CHRU de Nancy, Nancy, France; 2 INSERM U1116, Université de Lorraine, Nancy, France; 3 Service d’Anesthésie Réanimation de Chirurgie Cardiaque et Transplantation, Université de Lorraine, CHRU de Nancy, Nancy, France; 4 École de Chirurgie, Université de Lorraine, Nancy, France; 5 Service de Chirurgie Cardiaque, Université de Lorraine, Nancy, France; 6 Service de Biochimie, Université de Lorraine, Nancy, France; 7 Service d’Anatomie Pathologie, Laboratoires de Biologie Médicale et de Biopathologie, Université de Lorraine, Nancy, France

**Keywords:** Cardiac arrest, Veno-arterial extracorporeal membrane oxygenation, Vasoplegia

## Abstract

**Introduction:**

Post-cardiac arrest syndrome (PCAS) under veno-arterial ECMO (VA-ECMO) is frequently complicated by severe vasoplegia and microcirculation failure. Conventional resuscitation relies heavily on catecholamines and crystalloids loading, which may exacerbate endothelial dysfunction and oxidative stress. We evaluated whether a multimodal non-catecholaminergic strategy (vasopressin, methylene blue, albumin, and hypothermia) could improve hemodynamics and microcirculation compared to standard therapy with norepinephrine.

**Methods:**

Twenty pigs underwent surgically induced ischemic cardiac arrest and were resuscitated with VA-ECMO after 30 min of CPR. Animals were randomized (1:1) to receive either standard care (norepinephrine and crystalloids) or optimized treatment combining vasopressin, methylene blue, 20% albumin, and moderate hypothermia (34 °C). Hemodynamic, metabolic, and microcirculatory parameters were monitored for 6 h. Sublingual microcirculation was assessed using sidestream dark field (SDF) imaging.

**Results:**

Eighteen animals completed the protocol (9 per group). The optimized group required significantly less norepinephrine (7 mg [0–20] vs 78 mg [58–126], *p* = 0.0046) and fluid loading (4.5 L [4–5] vs. 14 L [13–18.5], *p* = 0.0007). Microcirculatory perfusion was markedly improved in the optimized group (PPV 88% vs. 28%, *p* < 0.001; MFI 2.25 vs. 0.25, *p* < 0.01). Lactate clearance did not differ significantly. Histological analysis showed less intestinal ischemic injury (Chiu/Park score 1 [0–1] vs. 4 [3–4], *p* = 0.003).

**Conclusion:**

In a porcine model of refractory cardiac arrest resuscitated with VA-ECMO, a multimodal non-catecholaminergic resuscitation strategy reduced vasopressor and fluid requirements and preserved microcirculation. These findings support multimodal, catecholamine-sparing approaches to limit endothelial dysfunction in ECMO-treated PCAS.

## Introduction

Out-of-hospital cardiac arrest (OHCA) remains a major global health challenge, with good neurological recovery (CPC 1–2) rarely exceeding 20% [1, 2]. Extracorporeal membrane oxygenation (ECMO) provides an effective means of mechanical circulatory support in refractory cardiac arrest and profound cardiogenic shock [3–5]. However, despite prompt restoration of systemic perfusion, survival remains limited by the post-cardiac arrest syndrome (PCAS), a complex state combining ischemia–reperfusion injury, systemic inflammation, and profound vasoplegia [6–9]. Following reperfusion, oxidative and nitrosative stress promote endothelial dysfunction, increased vascular leakage and microcirculatory collapse [10, 11]. Conventional resuscitation strategies—relying heavily on high-dose catecholamines and substantial crystalloid administration—may further exacerbate oxidative stress and impair microvascular flow [12–14]. These concerns have led to the emergence of “decatecholaminization” strategies, favoring non-adrenergic vasopressors and adjunctive therapies to restore vascular tone while limiting cellular injury.

Vasopressin acts via V1a receptors to induce vasoconstriction independent of adrenergic pathways, showing benefit in both septic and cardiogenic shock [15–17]. Methylene blue inhibits inducible nitric oxide synthase (iNOS) and guanylate cyclase, restoring vascular responsiveness [18–20]. Albumin contributes antioxidant and endothelial-stabilizing effects [21, 22], while therapeutic hypothermia attenuates metabolic stress and inflammation [23, 24]. Each of these interventions has shown favorable results in experimental models of shock or ECMO support; however, their combined effects have never been evaluated.

We hypothesized that a multimodal approach integrating vasopressin, methylene blue, albumin, and moderate hypothermia would improve hemodynamic stability, reduce catecholamine and fluid requirements, and preserve microcirculatory perfusion compared with standard norepinephrine-based therapy in a porcine model of refractory cardiac arrest resuscitated with VA-ECMO.

## Methods

### Study design and ethics

This prospective randomized controlled trial was conducted at the University of Lorraine surgical research facility (Nancy, France). All procedures complied with Directive 2010/63/EU and were approved by the institutional animal-ethics committee (APAFIS #40459–2023010518221016 v4). The study was reported in accordance with the ARRIVE 2.0 guidelines, and the corresponding checklist is provided as supplementary material (Supplementary File S1).

### Animal preparation

Twenty male Large-White pigs (50–55 kg) were acclimated for at least 7 days, fasted 12 h, and premedicated with ketamine 20 mg.kg^−1^ IM and midazolam 0.2 mg.kg^−1^. Anesthesia was induced with propofol 3 mg.kg^−1^ IV and maintained with propofol 10 mg.kg^−1^.h^−1^ and sufentanil 0.5 µg.kg^−1^.h^−1^; paralysis with cisatracurium (0.15 mg.kg^−1^ bolus then 0.06 mg.kg^−1^.h^−1^). Animals were mechanically ventilated (Dräger Evita XL) with tidal volume 8 mL.kg^−1^, FiO_2_ 0.5, PEEP 5 cmH_2_O. Targets were PaCO_2_ 35–45 mmHg and PaO_2_ 80–100 mmHg.

### Hemodynamic monitoring

Continuous ECG and rectal temperature monitoring were performed. A triple-lumen 8 Fr venous catheter (Arrow, USA) was inserted into the right external jugular vein. A 7 Fr arterial catheter (Seldicath, Plastimed Prodimed, France) was placed in the right carotid artery for continuous blood pressure monitoring. A bladder catheter (Cystofix, B. Braun, Germany) was inserted via lower midline laparotomy for hourly urine output measurement.

### ECMO circuit

After instrumentation, cannulation was performed. A 100 IU.kg^−1^ IV heparin loading dose (Heparin Sodique Choay, Sanofi-Aventis, France) was administered prior to cannulation, followed by continuous infusion at 50 IU.kg^−1^.h^−1^ to maintain an activated clotting time (ACT) of 180–250 s, monitored hourly (Hemochron Jr Signature, ITC, USA).

A ≥ 21 Fr venous drainage cannula (Biomedicus, Medtronic, USA) was inserted into the right atrium via sternotomy, and a ≥ 15 Fr arterial reinfusion cannula was percutaneously inserted into the right femoral artery using the Seldinger technique.

The ECMO system consisted of a Rotaflow console, centrifugal pump, tubing, and PLS-i oxygenator (all Maquet, Germany), connected to a mechanical gas blender (Sechrist Model 20090, USA). The circuit was primed with 0.9% saline. Sweep gas was adjusted repeatedly to maintain PaCO_2_ 35–45 mmHg and PaO_2_ 200–300 mmHg in oxygenator effluent.

### Cardiac arrest protocol

A median sternotomy and pericardiotomy were performed to expose the left anterior descending artery (LAD). Myocardial infarction was induced by proximal LAD ligation using a temporary tourniquet. Cardiac arrest occurred spontaneously approximately 3–5 min after LAD occlusion, typically presenting as ventricular fibrillation. LAD occlusion was maintained for a total of 60 min in order to reproduce a prolonged ischemic insult before reperfusion.

After 90 s of no-flow, resuscitation (low-flow) began. This no-flow duration is part of our standardized experimental cardiac arrest model and was chosen to reproduce the short delay typically observed between cardiac arrest onset and the initiation of resuscitation in clinical settings. For ventricular fibrillation, up to five internal defibrillation attempts were delivered, each followed by 2 min of internal cardiac massage. Cardiopulmonary resuscitation consisted of manual internal cardiac massage performed after sternotomy. Resuscitation quality was monitored using continuous end-tidal CO_2_ (EtCO_2_) monitoring. Mechanical chest compression devices were not used, as internal cardiac massage is the standard approach in our experimental model. Epinephrine (1 mg) and amiodarone (300 mg) were administered after the third shock and repeated as needed. In asystole, internal cardiac massage and epinephrine (1 mg every 2 min) were delivered up to five doses.

After 30 min of CPR, VA-ECMO was initiated at a fixed flow based on a theoretical cardiac output of 65–70 mL.kg^−1^.min^−1^. LAD occlusion was continued for 60 min after ECMO initiation, followed by reperfusion from H1 to H6. Animals were euthanized at the end of the experiment following sedation bolus and ECMO cessation.

Hemodynamic management after ECMO initiation: Mean arterial pressure (MAP) was maintained at ~ 65 mmHg using IV norepinephrine (Renaudin, France), where 1 mL contains 2 mg norepinephrine tartrate (equivalent to 1 mg norepinephrine base). Volume expansion with 0.9% saline was provided when cannula “chattering” suggested insufficient venous return and inadequate ECMO preload. Mean arterial pressure (MAP) was primarily controlled using norepinephrine, whereas fluid administration aimed to optimize venous drainage and maintain stable ECMO flow.

### Groups

Animals were randomized (1:1) into two groups at ECMO initiation:

i) Control group: managed conventionally with iterative crystalloid fluid resuscitation (NaCl 0.9%) and IV norepinephrine to maintain MAP > 65 mmHg.
ii) Experimental group named also optimized group: moderate hypothermia (target temperature 34 °C), initiated immediately after ECMO start, vasopressin (started at the dose of 0.01 IU.min^−1^and increased incrementally by 0.01 IU.min^−1^ to a maximum of 0.03 IU.min^−1^), methylene blue (bolus of 2 mg·kg^−1^ of intravenous MB (ProveBlue^®^, Provepharm, Marseille, France) perfused over the first 30 min after VA-ECMO initiation), and albumin fluid resuscitation (10 ml.kg^−1^ of 20% albumin solution (Human Albumin, 200 mg.ml^−1^, LFB^®^, France) up to 300 ml perfused over the first 3 h after a loading dose of 100 ml (maximum 400 ml infused). If MAP targets were not achieved, additional crystalloid resuscitation and norepinephrine support might be provided.

Timing of measurements: Hemodynamic measurements and biological assays were collected after surgery at baseline (HB), after refractory cardiac arrest (at ECMO initiation) (H0) and after three (H3) and six (H6) hours under ECMO. After H6 measurements, animals are euthanized with EXAGON^®^ (10 ml, Pentobarbital, Richter Pharma, Austria) following a sedation bolus and cessation of circulatory support.

Exclusion criteria: Animals were excluded if a major hemorrhagic event occurred before randomization, if cardiac arrest occurred before baseline measurements or if the ECMO device failed to provide the theoretical pump flow.

### Measured parameters

Hemodynamic measurements: Continuous monitoring of heart rate (HR), systolic (SAP), diastolic (DAP), and mean arterial pressure (MAP). Total fluid resuscitation volume and vasopressor rates were recorded at each time point. Urine output was continuously measured through the bladder catheter.

Laboratory measurements: Arterial blood gas was assessed in an acid–base and co-oxymeter analyzer at central temperature (VetStat, IDEXX Laboratories, France). Lactate concentration was measured using a biochemistry analyzer (VetStat, IDEXX Laboratories, France). Lactate clearance was calculated according to the following formula:

$${\mathrm{C}}\,_{{{\mathrm{LACTATE}}}} = \frac{{\left[ {{\mathrm{LACTATE}}\,{\mathrm{T0}}} \right] - \left[ {{\mathrm{LACTATE}}\,{\mathrm{T6}}} \right]}}{{\left[ {{\mathrm{LACTATE}}\,{\mathrm{T0}}} \right]}}\,\,\,\,\,{\mathrm{and}}\,{\mathrm{expressed}}\,{\mathrm{as}}\,\,{{\% }}$$

Microcirculation: The sublingual microvascular circulation was evaluated by a sidestream dark field (SDF) imaging device (Microscan, MicroVision Medical, Netherlands) according to published guidelines (12). The obtained video clips were analyzed semi-automatically using the Automated Vascular Analysis software (AVA 3.0 software, MicroVision Medical, Netherlands) from which the following microcirculation parameters were collected: total and perfused vessel density (TVD, PVD), proportion of perfused vessels (PPV) and microvascular flow index (MFI).

Norepinephrine–mean arterial pressure dose–response assessment: To establish the norepinephrine–mean arterial pressure dose–response curve, the norepinephrine infusion rate was increased stepwise (0, 1, 2, 3, 6, 9 and 10 µg·kg^−1^·min^−1^). At each step, the maximal increase in systolic, diastolic and mean arterial pressure was recorded after hemodynamic stabilization. This test was performed at H6 during ECMO support.

Histology: Ischemic enteric damage was histologically assessed. Proximal jejunum was sampled at the end of the experiment and fixed in a 10% formalin solution. Fixed tissues were embedded and sectioned for histological analysis. Slides were stained with hematoxylin, eosin and saffron (HES). The Park/Chiu scale, graded from 0 to 8, was used to assess the severity of ischemic digestive lesions.

After animal killing, the bloodless wet weight of the right lung was determined, and its dry weight was also measured after dehydration of the lobe during 24 h in an oven at 200 °C. The wet/dry weight ratio was used as an estimate of pulmonary vascular leakage.

### Statistical methods

This study was designed as an exploratory physiological study evaluating the effects of a multimodal non-catecholaminergic strategy in a controlled experimental ECMO model. The sample size was determined based on feasibility considerations and on previous large-animal ECMO studies with similar experimental designs. In accordance with ethical regulations for animal experimentation and the principles of the 3Rs (Reduction, Replacement, and Refinement), the number of animals was intentionally limited to the minimum required to obtain physiologically meaningful results.

The primary outcome was the norepinephrine requirement during the 6-h ECMO support period. Secondary outcomes included total fluid administration, microcirculatory parameters (TVD, PVD, PPV and MFI), lactate evolution and clearance, histological intestinal injury, and pulmonary vascular leakage assessed by the lung wet/dry weight ratio.

Results are presented as medians (interquartile range). Intergroup and intragroup differences over time were analyzed using a two-way repeated-measures ANOVA in order to account for repeated measurements during the experimental protocol. Data distribution was assessed before analysis. For comparisons between groups at specific time points, non-parametric Mann–Whitney tests were used. Statistical analyses were performed using GraphPad Prism 9 (GraphPad Software, USA), with *p* < 0.05 considered significant.

## Results

Twenty male Landrace pigs were included. Two animals (one per group) died before baseline measurements and were excluded. The remaining 18 pigs were randomized into the conventional group (*n* = 9) or the optimized group (*n* = 9). Baseline hemodynamic parameters were comparable between groups **(**Table 1**).**

**Table 1:** Baseline characteristics of the study population

Variables	Conventional group (*n* = 9)	Optimized group (*n* = 9)	*p*-value
Weight (kg)	53 [49–54]	51 [51–55]	0.92
Heart rate (bpm)	99 [77–100]	103 [93–126]	0.35
Temperature (°C)	38.0 [36.7–38.4]	38.1 [37.2–38.4]	0.69
Urine output (mL)	190 [150–250]	150 [100–400]	0.79
MAP (mmHg)	62 [55–70]	59 [55–74]	0.79
pH	7.40 [7.37–7.44]	7.33 [7.31–7.37]	0.07
PaCO_2_ (mmHg)	45 [4–45]*	53 [4–55]*	0.06
PaO_2_ (mmHg)	173 [166–186]	177 [167–197]	0.50
HCO_3_^−^ (mmol/L)	28 [28–32]	28 [28–30]	1.00
Lactate (mmol/L)	0.8 [0.6–1.2]	0.54 [0.49–1.50]	0.92

Data are presented as median [interquartile range]

*MAP*, mean arterial pressure; *PaCO₂*, arterial partial pressure of carbon dioxide; *PaO₂*, arterial partial pressure of oxygen; *HCO₃*^*−*^, bicarbonate concentration

### Model characterization

Characteristics at cardiac arrest are reported in Table S1. Time from LAD ligation to cardiac arrest was identical in both groups, and all animals presented with ventricular fibrillation as the initial rhythm. No differences were observed in no-flow or low-flow durations and return of spontaneous circulation (ROSC) was never achieved in any animal.

At ECMO initiation (H0), clinical and biological parameters were similar between groups except for temperature, which was lower in the optimized group (37.9 °C [37.1–38.3] vs. 35.8 °C [35.7–36.3], *p* = 0.008). Lactate levels at H0 were comparable (8.8 [7.95–11.1] mmol/L vs. 8.37 [7.51–10] mmol.l^−1^, *p* = 0.73). Baseline microcirculatory parameters also did not differ (Table S2).

### Hemodynamic parameters

Mean arterial pressure was continuously monitored and maintained close to the predefined target of approximately 65 mmHg in both groups throughout the experimental protocol (Supplementary Figure S1). Also, ECMO flow was achieved in both groups throughout the 6-h protocol.

**Fig. 1:**
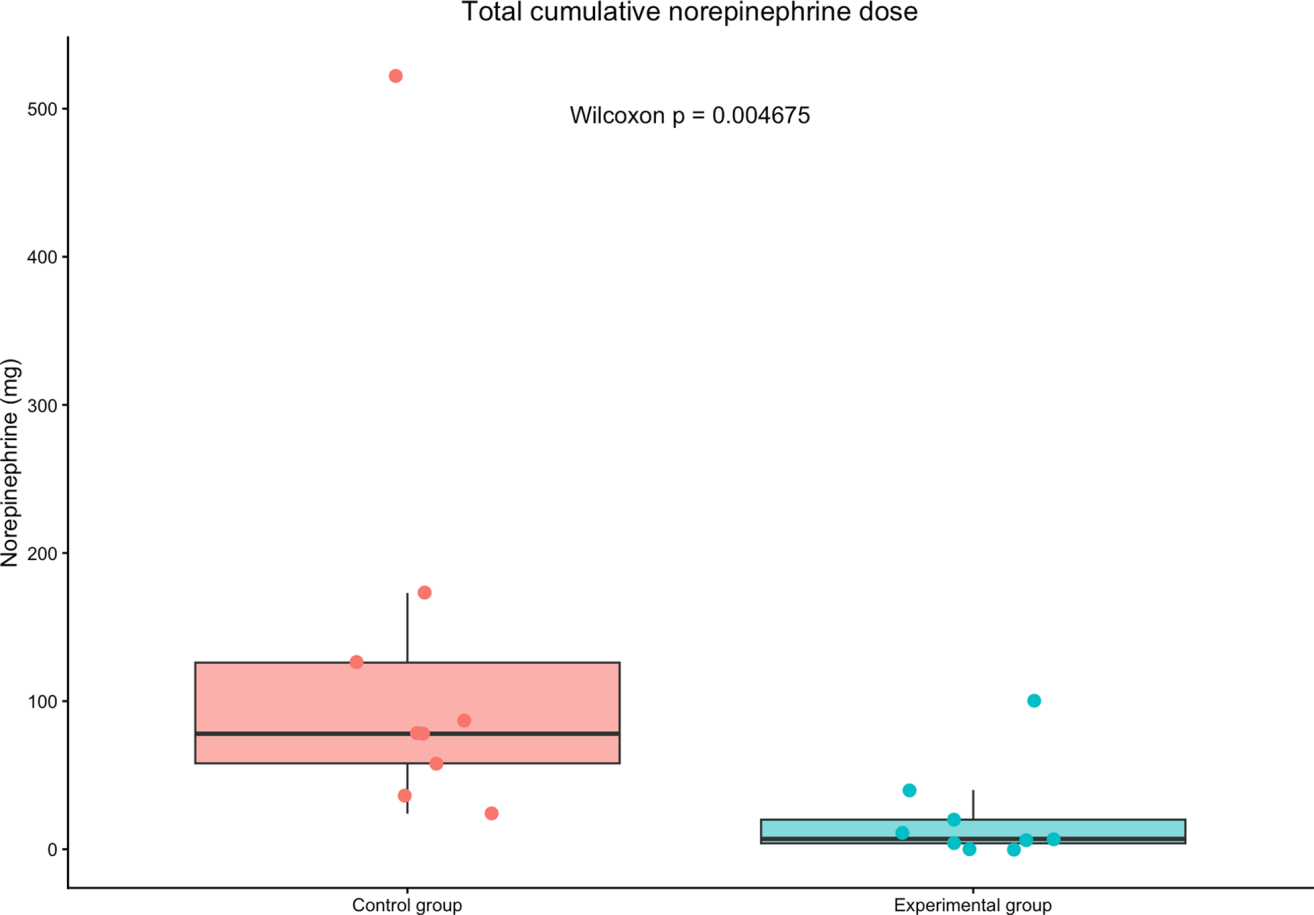
Total cumulative norepinephrine dose

The cumulative norepinephrine dose administered during the experiment was significantly higher in the conventional group compared with the optimized group (78 mg [58–126] vs 7 mg [0–20], *p* = 0.0046) **(**Fig. 1**).**

Urine output from H0 to H6 tended to be higher in the optimized group (445 ml [340–606] vs. 295 ml [185–330]), with a p-value at the threshold of significance (*p* = 0.05) (Figure S2).

**Fig. 2:**
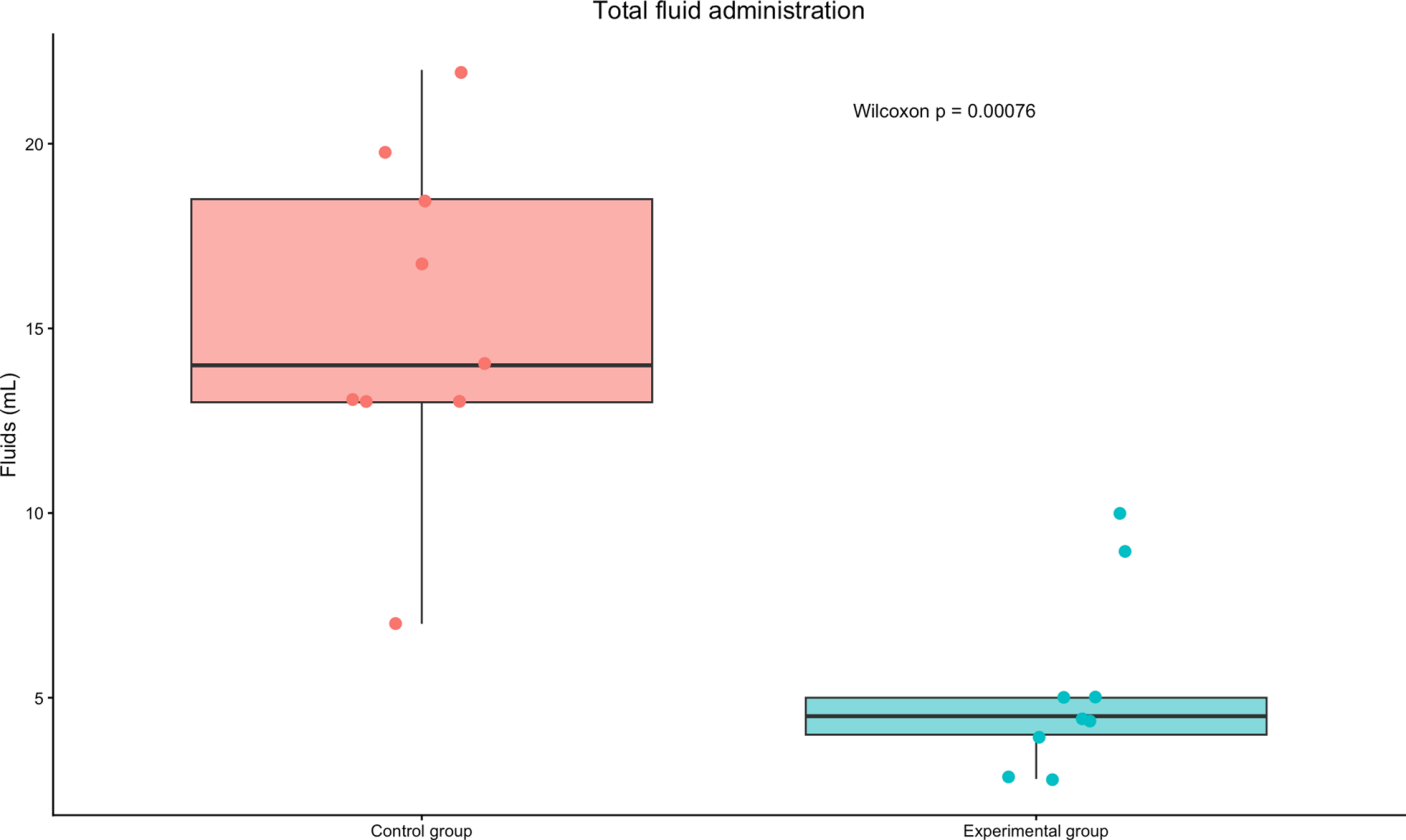
Total fluid administration

Fluid administration differed significantly between groups: 14 L [13–18.5] in the conventional group versus 4.5 L [4, 5] in the optimized group (*p* = 0.0007) (Fig. 2).

Vasoreactivity, evaluated through the norepinephrine dose–response curve, was significantly greater in the optimized group (p < 0.0001) (Figure S3).

### Lactate evolution

The median lactate levels at H6 were comparable between the two groups (11.7 [10.7–11.9] mmol.l^−1^ versus 11.2 [10.2–11.8] mmol.l^−1^; *p* = 0.59)**.** The median lactate clearance between H0 and H6 was negative due to the increase in lactate levels over time, despite both types of interventions. There was no significant difference between the two groups, with −27.8% [−46.36; −10.83] in the conventional group and −0.22% [−1.04; −0.16] in the optimized group (*p* = 0.53)**.** The evolution of lactate levels over time shows a trend toward lower lactate levels in the optimized group compared to the control group. However, this difference is not statistically significant (*p* = 0.13)**.**

### Microcirculatory parameters

Sublingual microcirculation was assessed using SDF imaging. TVD, PVD, PPV, and MFI were comparable at baseline (HB) and at H0 (Table S3).

TVD did not significantly differ between groups over time.

At H6, PVD, PPV, and MFI were significantly higher in the optimized group compared with the conventional group, indicating improved microvascular perfusion.

### Lung wet/dry weight ratio

The right lung wet/dry weight ratio was significantly lower in the optimized group (4.5 [4.5–5.14]) compared with the conventional group (5.29 [5.17–6.33], *p* = 0.03), indicating reduced pulmonary vascular leakage (Figure S4).

### Gut histology

Histological analysis revealed significantly less intestinal ischemic injury in the optimized group, with Chiu/Park scores of 1 [0–1] versus 4 [3, 4] in the conventional group (*p* = 0.003) (Figure S5).

## Discussion

This study demonstrates that a multimodal non-catecholaminergic resuscitation strategy combining vasopressin, methylene blue, albumin, and moderate hypothermia improves microcirculatory perfusion and limits endothelial injury in a porcine model of refractory cardiac arrest resuscitated with VA-ECMO. Despite similar macro-hemodynamic targets being achieved in both groups, the optimized strategy markedly reduced catecholamine and fluid requirements, indicating that targeting vascular function and endothelial integrity may enhance the quality of reperfusion beyond simple restoration of systemic arterial pressure.

### Mechanistic interpretation

Following global ischemia–reperfusion, profound vasoplegia results from nitric oxide overproduction, cGMP-mediated smooth muscle relaxation, mitochondrial dysfunction, and glycocalyx degradation [5, 25, 26]. The combination of vasodilation, impaired vasoreactivity, and capillary leak observed in PCAS parallels the pathophysiology of septic shock and postcardiotomy vasoplegic syndromes [10, 27, 28]. The therapeutic components evaluated here target complementary aspects of this dysfunction.

Vasopressin restores vascular tone via V1a receptor activation independently of adrenergic pathways, whereas methylene blue inhibits soluble guanylate cyclase and blunts the downstream consequences of nitric oxide excess, thereby improving vascular responsiveness [29]. Albumin contributes to endothelial protection by stabilizing the glycocalyx and scavenging reactive oxygen species [30, 31]. Moderate hypothermia limits reperfusion injury by reducing mitochondrial ROS formation, dampening cytokine release, and improving calcium homeostasis.

The combination of these agents therefore acts on both vascular tone and endothelial integrity, which may explain the robust improvements in microvascular perfusion observed. The marked increases in PVD, PPV, and MFI at H6 are consistent with the reductions in pulmonary permeability and intestinal ischemic injury, supporting a global protective effect on the endothelial barrier.

### Comparison with previous studies

Previous work in ECMO and shock models has reported beneficial effects of each component individually. In a cardiogenic shock pig model, Klein et al. [15] showed improved survival and lower lactate levels with vasopressin versus norepinephrine. Methylene blue has repeatedly demonstrated its ability to restore vascular reactivity in vasoplegia following cardiac surgery and in catecholamine-resistant septic shock [18, 19]. Albumin-based resuscitation has shown superior microcirculatory preservation compared with crystalloids in both hemorrhagic and septic shock [21, 22, 32]. Moderate hypothermia (33–34 °C) has improved myocardial recovery, reduced inflammatory cytokine levels, and attenuated reperfusion injury in ECMO-supported shock models [23, 24].

However, few studies have simultaneously combined these therapies in a clinically relevant model of ECMO-treated post-cardiac arrest syndrome. The present work extends prior findings by demonstrating additive or synergistic effects, particularly regarding microvascular flow—a critical determinant of organ recovery and outcome [33–35]. These results support the conceptual shift from pressure-oriented resuscitation toward strategies focused on vascular protection, perfusion quality, and endothelial health.

### Clinical implications

In clinical ECMO practice, high catecholamine exposure is associated with worse outcomes, likely through increased oxidative stress, microvascular dysfunction, arrhythmogenesis, and inflammatory activation [25, 36]. This experimental model suggests that early implementation of a multimodal catecholamine-sparing strategy is feasible and may substantially reduce adrenergic requirements. Introducing vasopressin and methylene blue early in the course of vasoplegia may help interrupt the cycle of refractory hypotension, capillary leak, and escalating norepinephrine doses.

The microcirculatory improvements observed here are particularly relevant, as persistent microvascular hypoperfusion after ECMO initiation has been independently associated with multiorgan failure and mortality. Optimizing capillary perfusion may therefore contribute to better organ recovery, even when macro-hemodynamic goals appear adequate. In addition, the combination of albumin with moderate hypothermia appears to limit endothelial injury and vascular leakage, a mechanism increasingly recognized as central in ECMO-related organ dysfunction [37, 38]. Angiotensin II was not included in the present multimodal strategy. Although this agent represents a promising non-adrenergic vasopressor in vasoplegic states, its interaction with the therapeutic bundle evaluated here remains unknown and deserves further investigation.

### Limitations

Several limitations must be acknowledged. First, the sample size was modest, and the study duration was limited to 6 h, preventing assessment of longer-term outcomes such as survival, neurological recovery, or delayed organ dysfunction. Second, as in all large-animal ECMO studies, interindividual variability in surgical instrumentation and perfusion management is possible, although randomization and standardized protocols mitigate this concern. Third, mechanistic analyses such as cytokine profiling, NO-related metabolites, or endothelial biomarkers were not performed, limiting the ability to delineate specific molecular pathways. Finally, healthy young pigs do not fully replicate the comorbidities, vascular heterogeneity, and inflammatory background of human ECMO patients.

Despite these limitations, the consistent effects observed across hemodynamic, microcirculatory, and histological endpoints reinforce the robustness of the findings and support further translational investigation.

## Conclusion

In this porcine model of refractory cardiac arrest supported by VA-ECMO, a multimodal non-catecholaminergic strategy combining vasopressin, methylene blue, albumin, and moderate hypothermia markedly reduced catecholamine and fluid requirements, improved microcirculatory perfusion, preserved endothelial integrity, and limited histologic organ injury. This catecholamine-sparing, endothelium-protective approach addresses key mechanisms of post-cardiac arrest vasoplegia and may enhance the quality of reperfusion under ECMO. These findings support further translational evaluation of multimodal catecholamine-sparing strategies to improve vascular function and organ perfusion during ECMO-supported post-cardiac arrest care.

## Data Availability

On reasonable request, by e-mail to Pr Levy, blevy5463@gmail.com.

## Abbreviations

ACT: Activated clotting time
AVA: Automated vascular analysis
CPR: Cardiopulmonary resuscitation
cGMP: Cyclic guanosine monophosphate
DAP: Diastolic arterial pressure
ECG: Electrocardiogram
ECMO: Extracorporeal membrane oxygenation
FiO₂: Fraction of inspired oxygen
HB: Baseline timepoint (pre-ECMO, “HB”)
H0/H3/H6: Measurements at ECMO initiation (H0), 3 h (H3), and 6 h (H6)
HCO₃⁻: Bicarbonate concentration
iNOS: Inducible nitric oxide synthase
IV: Intravenous
LAD: Left anterior descending artery
MAP: Mean arterial pressure
MB: Methylene blue
MFI: Microvascular flow index
NO: Nitric oxide
OHCA: Out-of-hospital cardiac arrest
PaCO₂: Arterial partial pressure of carbon dioxide
PaO₂: Arterial partial pressure of oxygen
PCAS: Post-cardiac arrest syndrome
PEEP: Positive end-expiratory pressure
PPV: Proportion of perfused vessels
PVD: Perfused vessel density
ROS: Reactive oxygen species
ROSC: Return of spontaneous circulation
SAP: Systolic arterial pressure
SDF: Sidestream dark field imaging
TVD: Total vessel density
VA-ECMO: Veno-arterial extracorporeal membrane Oxygenation
V1a: Vasopressin V1a receptor
VT: Tidal volume
W/D ratio: Lung wet-to-dry weight ratio
